# Complement-containing small extracellular vesicles from adventitial fibroblasts induce proinflammatory and metabolic reprogramming in macrophages

**DOI:** 10.1172/jci.insight.148382

**Published:** 2021-11-08

**Authors:** Sushil Kumar, Maria G. Frid, Hui Zhang, Min Li, Suzette Riddle, R. Dale Brown, Subhash Chandra Yadav, Micaela K. Roy, Monika E. Dzieciatkowska, Angelo D’Alessandro, Kirk C. Hansen, Kurt R. Stenmark

**Affiliations:** 1 Cardiovascular Pulmonary Research Laboratories, Departments of Pediatrics and Medicine, School of Medicine, University of Colorado Anschutz Medical Campus, Aurora, Colorado, USA.; 2 Department of Anatomy, All India Institute of Medical Sciences, New Delhi, India.; 3 Department of Biochemistry and Molecular Genetics, University of Colorado Anschutz Medical Campus, Aurora, Colorado, USA.

**Keywords:** Inflammation, Pulmonology, Cardiovascular disease, Hypertension, Macrophages

## Abstract

Pulmonary hypertension (PH) is a severe cardiopulmonary disease characterized by complement-dependent, fibroblast-induced perivascular accumulation and proinflammatory activation of macrophages. We hypothesized that, in PH, nanoscale-sized small extracellular vesicles (sEVs), released by perivascular/adventitial fibroblasts, are critical mediators of complement-dependent proinflammatory activation of macrophages. Pulmonary adventitial fibroblasts were isolated from calves with severe PH (PH-Fibs) and age-matched controls (CO-Fibs). PH-Fibs exhibited increased secretion of sEVs, compared with CO-Fibs, and sEV biological activity was tested on mouse and bovine bone marrow–derived macrophages (BMDMs) and showed similar responses. Compared with sEVs derived from CO-Fibs, sEVs derived from PH-Fibs (PH-Fib-sEVs) induced augmented expression of proinflammatory cytokines/chemokines and metabolic genes in BMDMs. Pharmacological blockade of exosome release from PH-Fibs resulted in significant attenuation of proinflammatory activation of BMDMs. “Bottom-up” proteomic analyses revealed significant enrichment of complement and coagulation cascades in PH-Fib-sEVs, including augmented expression of the complement component C3. We therefore examined whether the PH-Fib-sEV–mediated proinflammatory activation of BMDMs was complement C3 dependent. Treatment of PH-Fibs with siC3-RNA significantly attenuated the capacity of PH-Fib-sEVs for proinflammatory activation of BMDMs. PH-Fib-sEVs mediated proglycolytic alterations and complement-dependent activation of macrophages toward a proinflammatory phenotype, as confirmed by metabolomic studies. Thus, fibroblast-released sEVs served as critical mediators of complement-induced perivascular/microenvironmental inflammation in PH.

## Graphical abstract

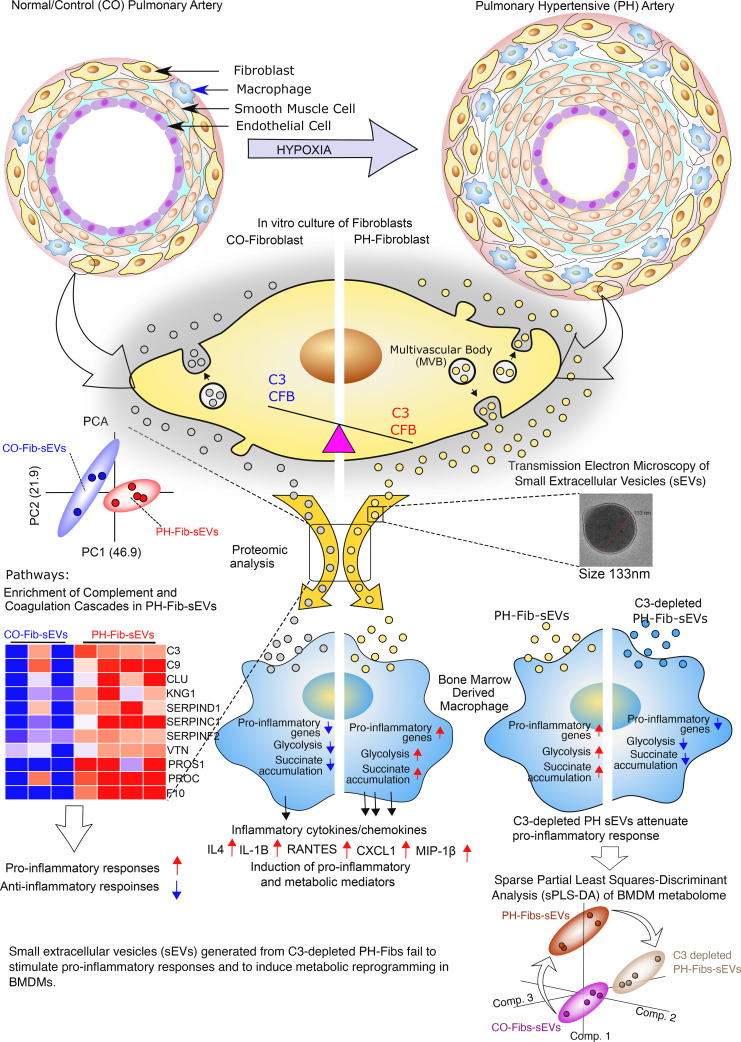

## Introduction

Pulmonary hypertension (PH) is a chronic and progressive cardiopulmonary disease associated with a sustained perivascular inflammation, severe vascular remodeling, and right heart failure (1–6). It is increasingly appreciated that inflammation, primarily characterized by early and persistent accumulation of perivascular macrophages, plays a critical role in vascular remodeling process associated with PH (2–4, 7–11). Recent studies have interrogated macrophage activation, relative to time and compartment (interstitial versus alveolar), using flow cytometric approaches in combination with RNA-Seq in hypoxic mouse models of PH (11, 12). Findings demonstrated that macrophage recruitment occurred early and was restricted to the interstitial/perivascular compartment, without recruitment of macrophages to the alveolar compartment or changes in the number of resident alveolar macrophages. Hypoxia-induced changes in the interstitial macrophages included mTORC1 signaling, metabolism, and activation of innate immune pathways, all well described with regard to hypoxia and PH (12). Although the role of innate immune pathways, including NF-κB, IL-1B, and IL-6 are well described with regard to their roles in PH, very little is known regarding their roles in the context of macrophages in PH, especially with regard to mechanisms of activation (3, 9).

In both the human condition and animal models, macrophage accumulation is largely perivascular in nature. Given that the fibroblast (Fib) is the principal resident cell in the perivascular compartment, our past studies have focused on Fib–macrophage interactions, believing this would provide insight into their specific interactions relevant to PH, especially because the majority of monocytes/macrophages present and contributing to the disease process are recruited, nonresident cells (11). Ex vivo studies have shown that vascular adventitial Fibs, obtained from humans with pulmonary arterial hypertension (PAH) and calves with severe hypoxia-induced PH, can recruit, retain, and activate monocytes/macrophages toward proinflammatory and proremodeling phenotype, at least in part, through soluble cytokine release (2, 12–15). Importantly, other studies have demonstrated that similar to macrophages in other injured tissues, proinflammatory activation of macrophages within the microenvironment of the injured artery wall in PH is associated with metabolic reprogramming characterized by aerobic glycolysis (16–18). The relevance of these studies is supported by our recent report, in which we showed that the proinflammatory and proremodeling phenotype of mouse bone marrow–derived macrophages (mBMDMs) treated with conditioned medium from cultured Fibs (control [CO] and PH) approximated the Inflammatory and metabolic phenotype of perivascular macrophages flow-sorted from the lungs of mice exposed to hypoxia in vivo (14).

Intercellular communication, a hallmark of vascular remodeling and inflammation, is thought to be mediated by both direct cell-to-cell contacts and paracrine mediators. Recent reports suggest that this intercellular communication is supported by the release of a broad range of mediators, including proteins, nucleic acids, lipids, signaling molecules, and metabolites via encapsulation in extracellular vesicles (EVs), that can go on to be integrated into the tissue and can modulate specific functions in target cells, including macrophage activation (7, 19–23). Recently, Khandagale et al. demonstrated that patients with PAH have higher levels of circulating EVs compared with healthy COs (24). To denote 3 different populations of EVs isolated by differential ultracentrifugation, the International Society for Extracellular Vesicles endorses use of the terms large EVs, medium EVs, and small EVs (sEVs; refs. 25, 26). To date, the majority of EV investigations have centered on sEVs. sEVs are nanoscale (50–150 nm in diameter) membrane-bound vesicles formed inside endosomal compartments that can act as positive or negative modulators of cardiovascular diseases depending on the type and state of the cells from which they originate (7, 19, 22, 25, 26). Recent experimental evidence demonstrates that EV-encapsulated cytokines are more stable than free cytokines (27). Osteikoetxea et al. found that exosomes are less sensitive to detergent lysis as they possess a liquid-ordered phase membrane (28). Among the EVs, sEVs have proven to be most stable and have been shown to stabilize their content and boost bioavailability of bioactive compounds (28, 29). Studies indicate that these vesicles are involved in a variety of critical cellular processes, including inflammation, immune response modulation, coagulation and disease progression (7, 22, 30).

The complement system, as the essential arm of innate immunity, has been shown to serve as an important interaction partner for EVs, and together they are associated with thrombotic and inflammatory conditions and appear to influence morbidity and mortality in several diseases (7, 30–32). Our recent work demonstrates that complement activation, and specifically that of the alternative pathway, plays a critical role in the early inflammatory and proliferative responses observed in PH (33). Complement activation was also noted to be important in the recruited macrophages that contributed to the PH process (11). Other studies have shown complex interactions between the complement system and EVs, with a dramatic influence on local and systemic inflammation and metabolic reprogramming (30, 31).

Although significant progress has been made in evaluating the microenvironment associated with PH/PAH, the mechanisms of vascular cell cross-talk through EVs, and specifically sEVs, are not known. The biological function of the sEVs can be directly elucidated by evaluation of their ability to influence cell signaling in the context of the proteomic signatures. In this study, we tested the hypothesis that sEVs, derived from perivascular/adventitial Fibs of animals with PH (PH-Fibs), but not from normal CO-Fibs, regulate proinflammatory and metabolic reprogramming responses in macrophages. We sought to determine whether sEVs derived from PH-Fibs (PH-Fib-sEVs), compared with sEVs derived from CO-Fibs (CO-Fib-sEVs), exhibited a distinct proteomic signature that contributed to macrophage activation toward a proinflammatory, metabolically primed phenotype. Differential profiles warranted evaluation of complement as an important determinant of the ability of sEVs to activate macrophages. Our data demonstrate that sEVs, released from adventitial Fibs of pulmonary hypertensive animals, acted as mediators of complement-driven activation of macrophages and may therefore have contributed to vascular remodeling and the progression of PH.

## Results

### Enrichment of sEVs in conditioned media of cultured Fibs, and sEV internalization by BMDMs.

**Figure 1:**
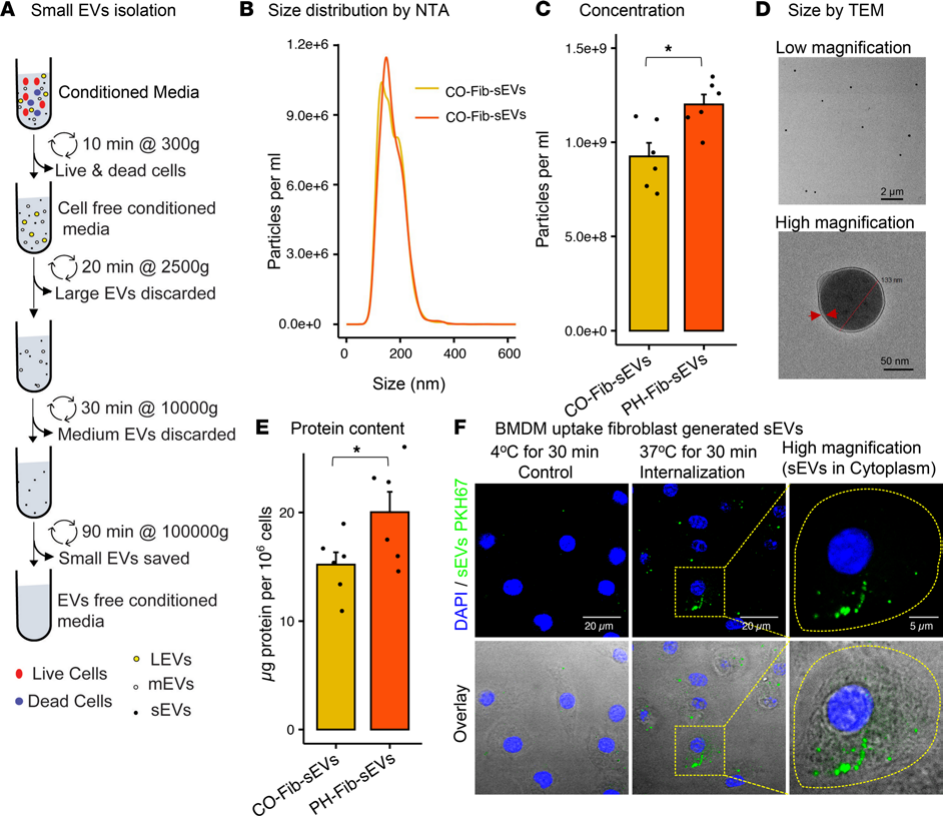
Evaluation of secretion of sEVs by CO-Fibs and PH-Fibs and assessment of sEV uptake by BMDMs. (**A**) Schematic diagram showing isolation of sEVs by differential ultracentrifugation from CM of cultured fibroblasts. (**B**) EVs (“particles”) isolated from pulmonary vascular adventitial CO-Fibs (*n* = 6) or PH-Fibs (*n* = 6) were analyzed for size and concentration in CM, using a NanoSight instrument and NTA. A “particle per mL” distribution plot demonstrates enrichment of EVs between 50 nm and 300 nm in size (sEVs). (**C**) Aggregate concentration of sEVs in CM demonstrated that PH-Fibs (*n* = 6) secreted a higher number of sEVs compared with CO-Fibs (*n* = 6). (**D**) Evaluation of the size and shape of sEVs with transmission electron microscopy. The high magnification image shows a double-membrane vesicle within the expected, for sEV, size range (133 nm). (**E**) sEVs isolated PH-Fib CM (*n* = 6) have higher protein content than sEVs from CO-Fib CM (*n* = 6). (**F**) BMDMs were incubated with PKH67-labeled sEVs at 37°C and at 4°C for 30 minutes. Confocal microscopy imaging (60×) demonstrates sEV internalization by BMDMs at 37°C, yet no uptake at 4°C. Data are presented as mean (**B**) and mean ± SEM (**C** and **E**). Unpaired *t* test with Welch’s correction was used for comparison between 2 groups. *P < 0.05. sEVs, small extracellular vesicles; BMDMs, bone marrow–derived macrophages; PH, pulmonary hypertension; PH-Fibs, fibroblasts of calves with severe PH; CO-Fibs, fibroblasts of age-matched controls; CM, conditioned medium; NTA, nanoparticle tracking analysis.

Vascular adventitial Fibs were isolated from CO or calves with PH, a well-established animal model of PH (34). We first sought to determine whether EVs were secreted by these Fibs and, if so, whether there were differences in the size or number of EVs from CO-Fibs versus PH-Fibs (Figure 1). Size distribution via nanoparticle tracking analysis (NTA) and overall concentration of EVs in conditioned media (CM) demonstrated enrichment of EVs between 50 nm and 300 nm, sEVs in particular (Figure 1B). PH-Fibs secreted a significantly greater number of sEVs per mL of CM, compared with CO-Fibs (Figure 1C). Further evaluation with transmission electron microscopy revealed double membrane vesicles within the expected range of size from 50 nm to 300 nm (Figure 1D). sEVs from PH-Fibs had a higher amount of total sEV protein per 10^6^ cells than those from CO-Fibs (Figure 1E). To assess whether BMDMs internalize Fib-derived sEVs, we labeled sEVs with the lipophilic dye PKH-67 and incubated them with mBMDMs for 30 minutes. Confocal microscopy imaging of BMDMs confirmed sEV internalization (Figure 1F).

### sEVs derived from PH-Fibs mediated BMDM activation toward a proinflammatory phenotype.

**Figure 2:**
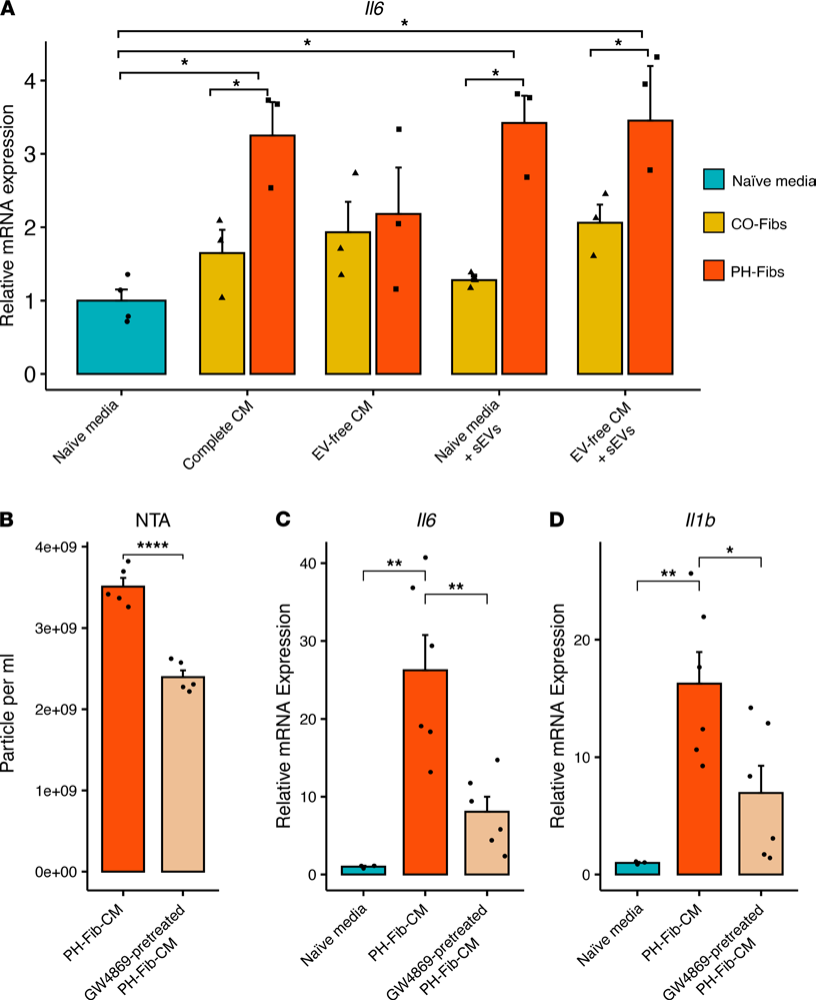
sEVs contribute, in large part, to promoting the proinﬂammatory effect of PH-Fib-CM on BMDMs. (**A**) sEVs and complete CM from PH-Fibs (*n* = 3) elicited increased *Il6* mRNA expression in BMDMs, compared with “naive” media, whereas the effect of CM depleted of EVs (EV-free CM), or CO-Fibs complete CM or sEVs (*n* = 3), were not statistically significant. (**B**) NTA confirms that GW4869, an inhibitor of EV secretion, inhibited release of EVs by PH-Fibs into CM. (**C** and **D**) BMDMs incubated with CM from GW4869-pretreated PH-Fibs (*n* = 6) had significantly attenuated *Il6* (**C**) and *Il1b* (**D**) expression, compared with BMDMs incubated with CM from untreated PH-Fibs (*n* = 6). Data are presented as mean ± SEM. Two-way ANOVA (**A**) or 1-way ANOVA (**C** and **D**) followed by Tukey’s multiple-comparison test were performed. Unpaired *t* test with Welch’s correction (**B**) was used for comparison between 2 groups. *P < 0.05, **P < 0.01, ****P < 0.0001. sEVs, small extracellular vesicles; BMDMs, bone marrow–derived macrophages; PH, pulmonary hypertension; PH-Fibs, fibroblasts of calves with severe PH; CO-Fibs, fibroblasts of age-matched controls; CM, conditioned medium; NTA, nanoparticle tracking analysis.

To determine which component(s) of the fibroblast CM (Fib-CM) can activate BMDMs, we analyzed several fractions (shown in Figure 1A) of Fib-CM and *Il6* gene expression as a readout of activation (13, 34). Both complete CM and sEVs from PH-Fibs elicited increased expression of the *Il6* gene in BMDMs, whereas EV-free CM or sEVs from CO-Fibs did not (Figure 2A). To further substantiate that sEVs are an important component of the effects of PH-Fib-CM on macrophage activation (defined by *Il6* and *Il1b* expression), we determined the effect(s) of treatment of PH-Fibs with GW4869, an inhibitor of EV secretion (35). GW4869 was found to decrease the number of EVs released by PH-Fibs, and the stimulatory effects of PH-Fib-CM on *Il6* and *Il1b* expression in BMDMs was significantly attenuated (Figure 2, B–D).

**Figure 3:**
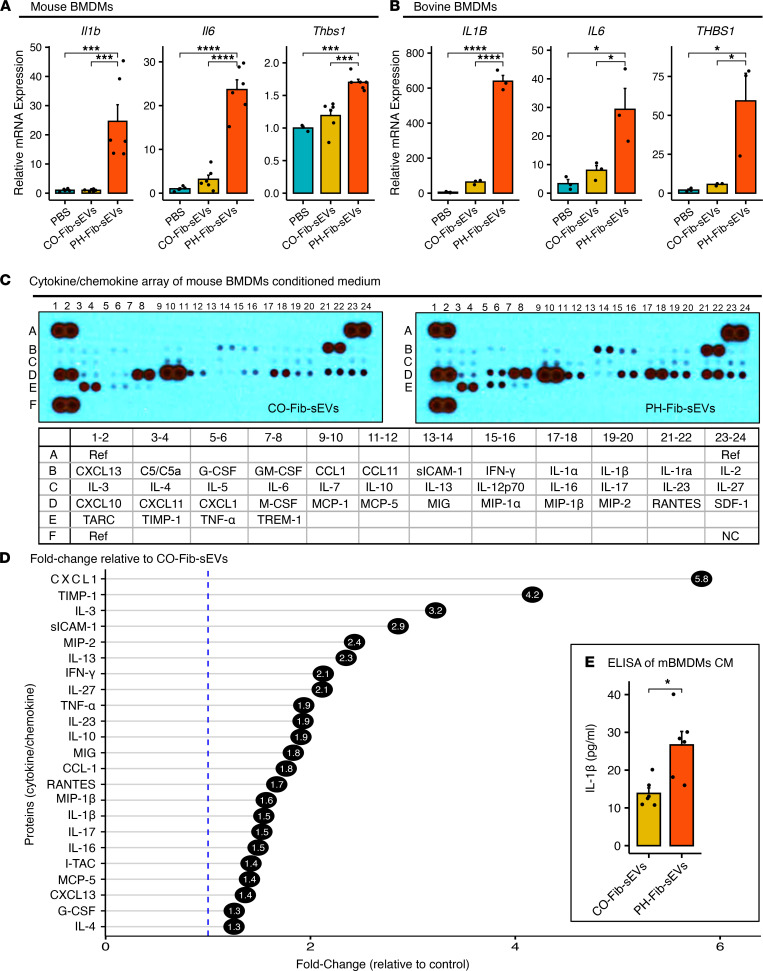
sEVs derived from PH-Fibs, but not from CO-Fibs, mediate BMDM activation toward a proinﬂammatory phenotype. (**A**) Mouse BMDMs incubated with sEVs from PH-Fibs, but not those from CO-Fibs (*n* = 6, each group), significantly upregulated *Il1b*, *Il6*, and *Thbs1* expression. (**B**) Bovine BMDMs, incubated with (autogenic) sEVs from PH-Fibs, but not CO-Fibs (*n* = 3, each group), profoundly (note the scale differences between **A **and **B**) upregulated *IL1B*, *IL6*, and *THBS1* expression. (**C** and **D**) Mouse BMDMs were incubated with sEVs from CO-Fibs or PH-Fibs for 16 hours; then culture supernatant was collected and subjected to Mouse Cytokine/Chemokine Array analysis (**C**), where the table (lower panel) shows the detected analytes and their location on the membrane. (**D**) The data obtained from (**C**) were quantified using NIH ImageJ software, and mean pixel density was normalized to the density of the respective positive controls. Relative expression of cytokines was computed, and the fold change of PH-Fib-sEVs, relative to CO-Fib-sEVs, is shown. (**E**) IL-1β released by BMDMs incubated with CO-Fib-sEVs or PH-Fib-sEVs was confirmed by ELISA. Data are presented as mean ± SEM (**A**, **B**, and **E**) and mean (**D**). One-way ANOVA (**A** and **B**) followed by Tukey’s multiple-comparison test was performed. Unpaired *t* test with Welch’s correction (**E**) was used for comparison between 2 groups. *P < 0.05, ***P < 0.001, ****P < 0.0001. sEVs, small extracellular vesicles; BMDMs, bone marrow–derived macrophages; PH, pulmonary hypertension; PH-Fibs, fibroblasts of calves with severe PH; CO-Fibs, fibroblasts of age-matched controls.

After the confirmation that PH-Fib–derived sEVs alone stimulated cytokine expression in BMDMs, we evaluated their effects on other inflammation-related genes. PH-Fib-sEVs significantly induced expression of proinflammatory cytokines *Il6*, *Il1b*, and *Thbs1*, in mBMDMs, and even more prominently in bovine (autogenic) BMDMs (Figure 3, A and B) compared with CO-Fibs or PBS. To investigate whether Fib-derived sEVs induced release of inflammatory proteins from BMDMs to the microenvironment, we treated mBMDMs with sEVs from CO-Fibs or PH-Fibs and evaluated the culture supernatant via cytokine array. As demonstrated in Figure 3, C and D, BMDMs treated with PH-Fib-sEVs compared with CO-Fib-sEVs released higher levels of inflammatory cytokines and chemokines to culture medium, including IL-4, G-CSF, BLC (CXCL13), MCP-5, I-TAC (CXCL11), IL-16, IL-17, IL-1β, MIP-1β, RANTES, I-309 (CCL1), MIG, IL-10, IL-23, TNF-α, IL-27, IFN-γ, IL-13, MIP-2, sICAM-1, IL-3, TIMP-1, and CXCL-1. ELISA-based quantification of IL-1β production confirmed that BMDMs treated with PH-Fib-sEVs induced higher release of IL-1β than those treated with CO-Fib-sEVs (Figure 3E).

### sEVs derived from PH-Fibs induced marked changes in the metabolic state of BMDMs.

**Figure 4:**
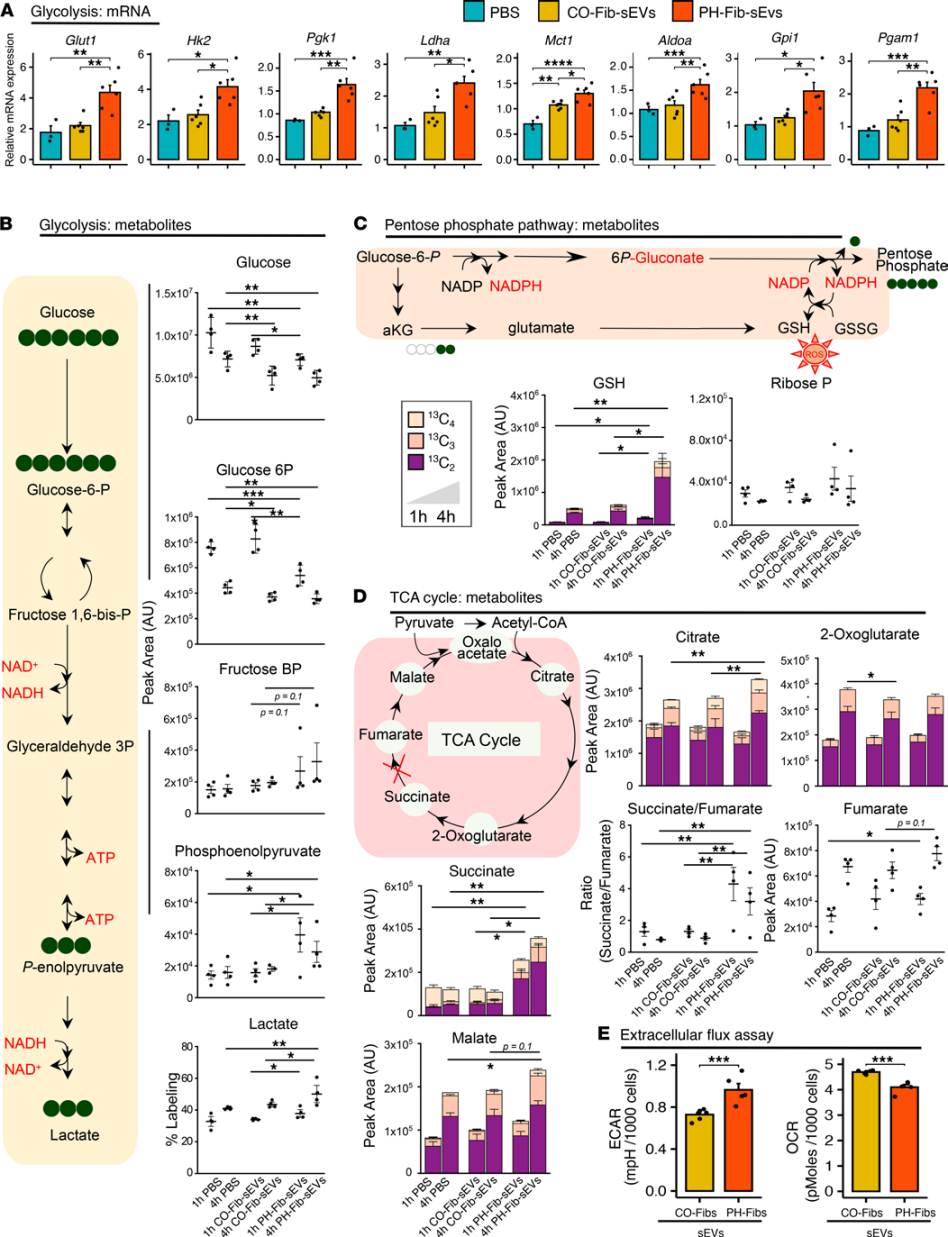
sEVs derived from PH-Fibs mediate metabolic reprogramming of mouse BMDMs. (**A**) sEVs from PH-Fibs (*n* = 6) significantly upregulated expression of *Glut1*, *Hk2*, *Pgk1*, *Ldha*, *Mct1*, *Aldoa,* and *Pgam1* mRNA in BMDMs. (**B**–**D**) BMDMs were incubated with PBS, or with sEVs from CO-Fibs or PH-Fibs (*n* = 4, each group), in the presence of stable isotope-labeled U-^13^C_6_-glucose for 1 hour and 4 hours. Labeled carbon tracing in glycolysis (**B**), PPP and glutathione synthesis (**C**), and the TCA cycle (**D**) directly confirmed a significant effect of PH-Fib–derived sEVs in promoting glucose oxidation via glycolysis (especially evident when considering isotopologue distributions for labeled lactate), but not on the PPP. Significant accumulation of labeled succinate and ^13^C_2_-succinate/^13^C_2_-fumarate ratios is likely indicative of a metabolic blockade at the level of complex II in mitochondria. (**E**) Analyses of ECAR and OCR were performed in Seahorse XFe96 analyzer to assess glycolysis and mitochondrial respiration. BMDMs treated with PH-Fib-sEVs (*n* = 6), compared with those treated with CO-Fib-sEVs (*n* = 6), demonstrated higher ECAR and lower OCR. (**A** and **E**) Data are presented (*n* = 6, each group) as mean ± SEM. (**B**–**D**) Data are presented (*n* = 4 each group) as mean ± SEM. (**A**–**D**) One-way ANOVA followed by Tukey’s multiple-comparison test was performed. Unpaired *t* test with Welch’s correction (**E**) was used for comparison between 2 groups. *P < 0.05, **P < 0.01, ***P < 0.001, ****P < 0.0001. sEVs, small extracellular vesicles; BMDMs, bone marrow–derived macrophages; PH, pulmonary hypertension; PH-Fibs, fibroblasts of calves with severe PH; CO-Fibs, fibroblasts of age-matched controls; PPP, pentose phosphate pathway; ECAR, extracellular acidification rate; OCR, oxygen consumption rate.

Next, the effects of sEVs derived from CO-Fibs or PH-Fibs on metabolic gene expression by both mouse and bovine BMDMs were evaluated. We observed marked increases in the expression of metabolic genes, including *Glut1*, *Hk2*, *Gpi1*, *Aldoa*, *Pgk1*, *Pgm1*, *Ldha*, and *Mct1*, in mBMDMs in response to PH-Fib-sEVs compared with CO-Fib-sEVs (Figure 4A). Similar findings were observed in bovine BMDMs (Supplemental Figure 1; supplemental material available online with this article; https://doi.org/10.1172/jci.insight.148382DS1).

To validate these findings and directly test the effect of sEVs on the metabolism of BMDMs, we performed tracing experiments with U-^13^C_6_-glucose for 1 hour and 4 hours. Results indicated a significant effect of sEVs from PH-Fibs on labeled glucose consumption, consumption of labeled hexose phosphate compounds (including glucose 6-phosphate), and accumulation of fructose bisphosphate (*P* = 0.1), phosphoenolpyruvate, and lactate (Figure 4B). In particular, labeled lactate represented a significantly higher percentage of total isotopologues in BMDMs incubated with PH-Fib-sEVs compared with CO-Fib-sEVs or PBS CO (Figure 4B). On the other hand, no significant changes in labeled ribose phosphate levels were observed across the 3 groups, suggesting a lack of significant alterations to the pentose phosphate pathway (Figure 4C). De novo synthesis of reduced glutathione, an adenosine triphosphate–dependent process, was instead increased in BMDMs treated with PH-Fib-sEVs (Figure 4C). Interestingly, the labeling scheme is consistent with increased glutathione synthesis from ^13^C_2_-glutamate, which is derived from transamination of ^13^C_2_-2-oxoglutarate (Figure 4D). Notably, despite increases in labeled citrate isotopologues, PH-Fib-sEVs did not induce significant accumulation of labeled 2-oxoglutarate (Figure 4D). Strikingly, ^13^C-succinate accumulated significantly in the PH-Fib-sEV group at 1 hour and 4 hours, with succinate accumulation occurring at a much faster rate than downstream carboxylic acids, fumarate, and malate (Figure 4D). Indeed, ^13^C_2_-succinate/^13^C_2_-fumarate ratios were significantly higher in BMDMs treated with PH-Fib-sEVs compared with those treated with CO-Fib-sEVs or PBS (Figure 4D), a finding consistent with BMDM proinflammatory state (34).

Furthermore, the profiles of mitochondrial respiration and glycolysis, using the Seahorse extracellular flux analyzer (Seahorse XFe96), of BMDMs treated with sEVs from CO-Fibs or PH-Fibs also confirmed that PH-Fib-sEVs stimulated glycolysis (extracellular acidification rate) in BMDMs (Figure 4E, left). However, oxidative respiration (oxygen consumption rate) of BMDMs in response to PH-Fib-sEVs was low compared with the CO group (Figure 4E, right).

### sEVs derived from PH-Fibs expressed a distinct proteomic signature.

**Figure 5:**
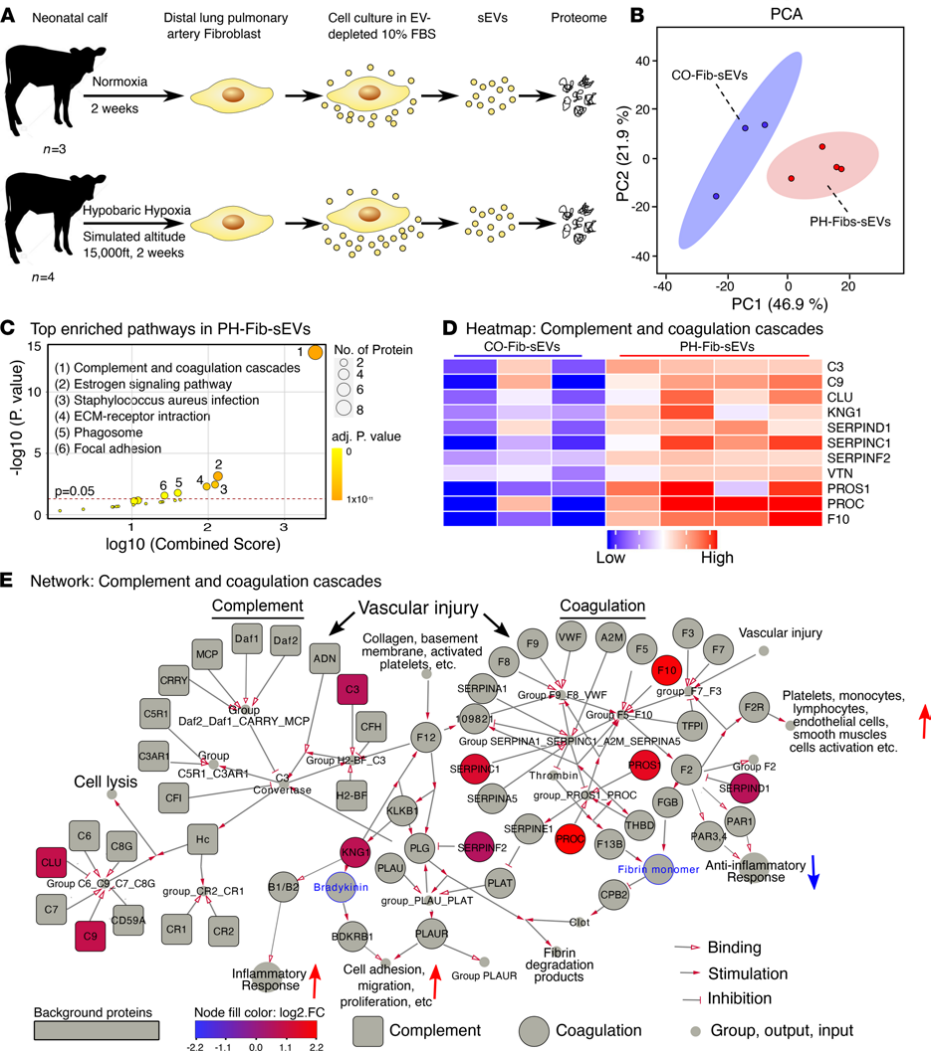
Proteomic analysis of sEVs from CO-Fibs and PH-Fibs shows broad and distinct proteomic signatures, wherein protein content of PH-Fib-sEVs demonstrates enrichment of complement and coagulation cascades. (**A**) Schematic diagram showing experimental methodology. (**B**) Principal component analysis of protein content of sEVs demonstrates a distinct variation of PH-FIb-sEVs versus CO-Fib-sEVs. Ellipses around each group of samples represent 95% CI. (**C**) Bubble plot of pathway enrichment analysis of protein content of sEVs derived from PH-Fibs compared with CO-Fibs shows (a) a very significant enrichment of complement and coagulation cascades as well as of (b) estrogen signaling pathway, (c) staphylococcus aureus infection, (d) ECM receptor interaction, (e) phagosome, and (f) focal adhesion pathway. (**D**) Heatmap demonstrates significantly upregulated proteins in PH-Fib–derived sEVs that are associated with activation of complement and coagulation cascades: complement components C3 and C9 as well as clusterin and coagulation regulators, KNG1, SERPIND1, SERPINC1, SERPINF2, VTN, PROS1, PROC, and F10. These proteins were significantly upregulated in sEVs from PH-Fibs (Supplemental Table 1). (**E**) The network of protein interactions of complement (shown in squares) and coagulation (shown in circles) cascades was generated from KEGG database, Wiki pathways, and Reactome databases. Significantly upregulated proteins in PH-Fib-sEVs are shown in red, and metabolites are defined in blue text. The “up” pointing red arrows define activation and “down” pointing blue arrows define inhibition. sEVs, small extracellular vesicles; BMDMs, bone marrow–derived macrophages; PH, pulmonary hypertension; PH-Fibs, fibroblasts of calves with severe PH; CO-Fibs, fibroblasts of age-matched controls.

Because PH-Fib-sEVs, but not CO-Fib-sEVs, activated BMDMs toward a proinflammatory phenotype and induced their metabolic reprogramming, we next evaluated the protein content of PH-Fib-sEVs using high-resolution mass spectrometry (UHPLC-MS/MS; Figure 5A). An unsupervised data reduction approach was used to reveal differences between PH-Fib-sEVs and CO-Fib-sEVs (principal component analysis [PCA]; Figure 5B). Heatmaps and volcano plots also confirmed expression of sEVs/exosome markers (ALIX, CD9, CD81), as well as differentially expressed proteins (Supplemental Figure 2, A and B, and Supplemental Table 1). Collectively, the data demonstrate that PH-Fib–derived sEVs exhibited a distinct proteomic signature compared with CO-Fib-sEVs.

### Gene ontology and pathway enrichment analyses of differentially expressed genes demonstrated the activation of the complement and coagulation cascades in PH-Fib-sEVs.

Gene ontology (GO) analysis of the sEV proteomes is shown in Supplemental Figure 3. We focused on 2 aspects of the GO profile. The cellular component of differentially expressed sEV proteins revealed enrichment of EV and exosome, granule and plasma membrane, cytoskeleton, and lipoprotein-related GO in PH-Fibs (Supplemental Figure 3A). Next, we assessed the biological processes of sEVs. GO analysis of the biological processes of PH-Fib-sEV proteins demonstrated overrepresentation of immune response, response to wounding, inflammation response, biological regulation, and biological adhesion, including activation of complement (Supplemental Figure 3B). Pathway enrichment analysis of proteins in PH-Fib-sEVs showed profound enrichment in complement and coagulation cascade pathways (Figure 5C). In addition, estrogen signaling pathway, ECM-receptor interaction, phagosome, and focal adhesion pathways were also significantly enriched in the PH-Fib-sEV group (Figure 5C). Next, we examined the protein profiling of complement and coagulation cascades. We found that complement components C3, C9, and clusterin as well as coagulation regulators (KNG1, SERPIND1, SERPINC1, SERPINF2, VTN, PROS1, PROC, F10) were significantly upregulated in PH-Fib-sEVs (Figure 5D). The network of complement and coagulation cascades (Figure 5E), initiated by vascular injury and other factors, showed that upregulated proteins in PH-Fib-sEVs can induce (a) cell adhesion, migration, and proliferation (36); (b) platelet, monocyte, lymphocyte, endothelial, and smooth muscle cell activation (36); (c) inflammatory response (36, 37); and (d) inhibition of antiinflammatory response (38) to target cells (BMDMs). Moreover, C3 can also induce an inflammatory response via C3ar1/C5ar1 axes in target cells (complement network in Figure 5E).

### sEVs from C3-depleted PH-Fibs failed to stimulate BMDM inflammatory gene expression.

**Figure 6:**
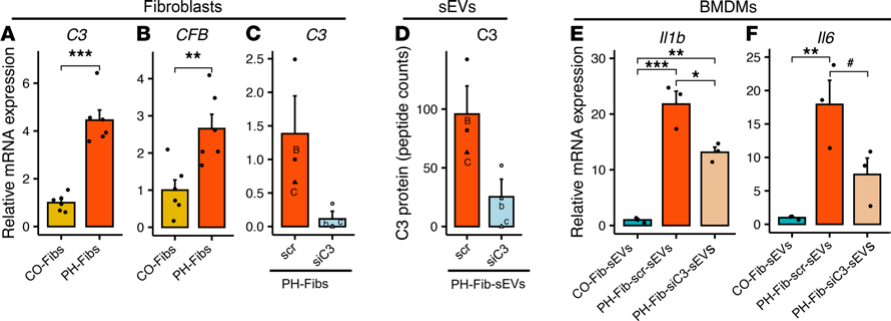
sEVs from PH-Fibs, depleted of complement C3, exhibit significantly attenuated proinﬂammatory effect on BMDMs. (**A** and **B**) mRNA expression levels of complement components *C3* and *CFB* were high in PH-Fibs compared with CO-Fibs (*n* = 6, each group). (**C** and **D**) siRNA-mediated complement C3 knockdown in PH-Fibs (*n* = 3) resulted in very low *C3* mRNA expression in PH-Fibs (**C**), and markedly attenuated C3 protein expression in PH-Fib–derived sEVs (**D**). (**E** and **F**) BMDMs incubated with sEVs derived from PH-Fibs transfected with scramble siRNA exhibited markedly upregulated *Il1b* and *Il6* expression, whereas BMDMs incubated with sEVs, derived from siC3-treated PH-Fibs, showed significantly attenuated expression of *Il1b* and *Il6*. Data are presented as mean ± SEM. (**A**–**D**) Unpaired *t* test with Welch’s correction was used for comparison between 2 groups. (**F**) One-way ANOVA followed by Tukey’s multiple-comparison test was performed. ^#^*P* = 0.0582, *P < 0.05, **P < 0.01, ***P < 0.001. sEVs, small extracellular vesicles; BMDMs, bone marrow–derived macrophages; PH, pulmonary hypertension; PH-Fibs, fibroblasts of calves with severe PH; CO-Fibs, fibroblasts of age-matched controls.

To determine whether changes in intracellular complement signaling contribute to complement-mediated effects of PH-Fib-sEVs, we examined gene expression of complement C3 and factor B (CFB), the 2 major components of the alternative activation pathway, in Fibs. The mRNA expression levels of *C3* and *CFB* were significantly increased in PH-Fibs compared with CO-Fibs (Figure 6, A and B). Next, to evaluate whether increased C3 expression in PH-Fibs directly correlated with C3 protein levels in PH-Fib-sEVs, we knocked down C3 in PH-Fibs, employing siC3 RNA, and used scramble (scr) RNA-Seq as a CO (Figure 6C). We then isolated sEVs from the CM of siC3-transfected PH-Fibs and confirmed C3 protein depletion (Figure 6D and Supplemental Figure 4). We examined the effects of sEVs from siC3-transfected PH-Fibs (siC3-PH-Fib-sEVs) and scr-transfected PH-Fibs (scr-PH-Fib-sEVs) on BMDMs and observed that scr-PH-Fib-sEVs did induce expression of *Il1b* and *Il6* in BMDMs, whereas siC3-PH-Fib-sEVs had a significantly attenuated capacity to induce *Il1b* and *Il6* gene expression (Figure 6, E and F).

### sEVs from C3-depleted PH-Fibs failed to induce metabolic reprogramming in BMDMs.

**Figure 7:**
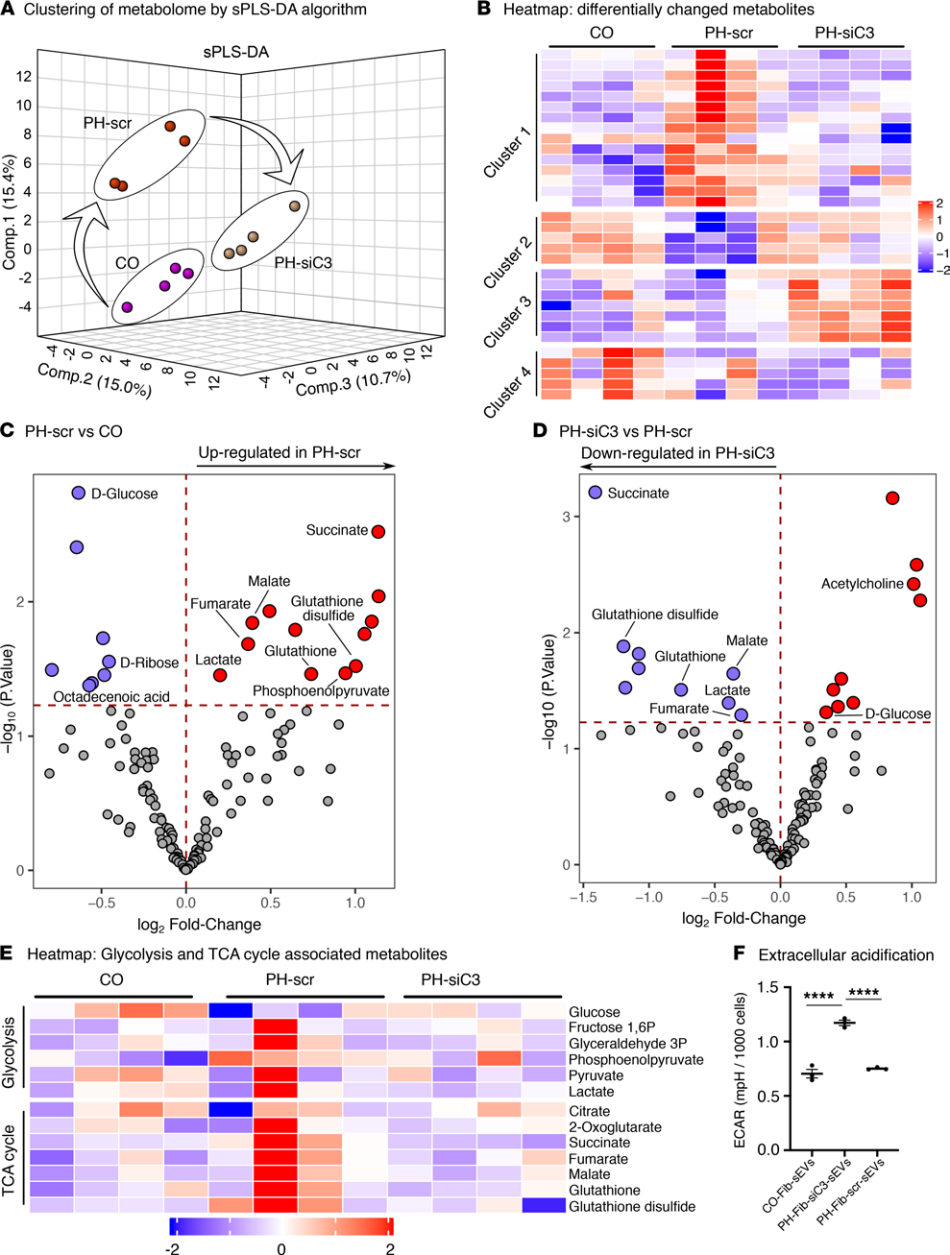
sEVs from PH-Fibs, depleted of complement C3, exhibit significantly attenuated metabolic reprogramming of BMDMs. (**A**) Clustering, performed by sparse partial least squares discriminant analysis of metabolites from BMDMs treated with sEVs derived either from CO-Fibs (CO, *n* = 4) or PH-Fibs transfected with scr-siRNA (PH-scr, *n* = 4) or from siC3-transfected PH-Fibs (PH-siC3, *n* = 4) demonstrate profoundly distinct changes. Notably, the BMDM group treated with sEVs from siC3-PH-Fibs is closer to CO than the PH-scr group. (**B**) Heatmap of metabolites of BMDMs treated with sEVs from CO-Fibs (CO), scr-PH-Fibs (PH-scr), or siC3-transfected PH-Fibs (PH-siC3) confirms the differences and similarities among the 3 groups shown in **A**. (**C** and **D**) Volcano plots of differential changes in BMDM metabolites demonstrate significant upregulation and downregulation, wherein horizontal dashed lines represent *P* cutoff = 0.05, and thus the changes in metabolites shown above these lines are statistically significant (Supplemental Table 2). The metabolites shown in the upper left compartments represent those that are significantly downregulated, whereas the metabolites shown in the upper right compartments represent those that are significantly upregulated. (**E**) The heatmap of glycolysis and TCA cycle represents the levels of metabolites in individual samples. (**F**) Analysis of ECAR was performed using the Seahorse XFe96 analyzer to assess glycolysis. ECAR was markedly higher in BMDMs treated with sEVs from scr-siRNA–transfected PH-Fibs, compared with BMDMs treated with CO-Fib-sEVs. BMDMs incubated with sEVs from siC3-transfected PH-Fibs exhibited the same ECAR as CO-Fib-sEV–treated BMDMs. (**C** and **D**) Data are presented (*n* = 4 each group) as mean. (**F**) Data are presented (*n* = 3 each group) as mean ± SEM. One-way ANOVA followed by Tukey’s multiple-comparison test was performed. ****P < 0.0001. sEVs, small extracellular vesicles; BMDMs, bone marrow–derived macrophages; PH, pulmonary hypertension; PH-Fibs, fibroblasts of calves with severe PH; CO-Fibs, fibroblasts of age-matched controls; ECAR, extracellular acidification rate; scr, scramble.

To evaluate the complement-mediated effects of sEVs on the metabolic state of BMDMs, we performed LC-MS analysis of small molecules extracted from BMDMs that were treated with siC3-PH-Fib-sEVs or scr-PH-Fib-sEVs. Analysis of metabolites showed that BMDMs treated with sEVs from siC3-PH-Fibs remained in a state comparable to the cells treated with CO-Fib-sEVs. Both of these groups exhibited a very different metabolic profile compared with BMDMs treated with scr-PH-Fib-sEVs (Figure 7A). A heatmap of all the significantly (*P* < 0.05) altered metabolites confirmed the clustering by sPLS-DA and showed that siC3-PH-Fib-sEVs exhibited metabolic reprogramming rather similar to that of CO-Fib-sEVs (Figure 7B). In contrast, scr-PH-Fib-sEVs induced succinate, malate, fumarate, glutathione, and glutathione disulfide metabolite production, whereas these metabolites were all downregulated in BMDMs treated with siC3-PH-Fib-sEVs (Figure 7, C and D, and Supplemental Table 2, A and B). Succinate was the most upregulated metabolite in BMDMs treated with scr-PH-Fib-sEVs and the most downregulated metabolite in BMDMs treated with siC3-PH-Fib-sEVs (Figure 7, C–E). We next confirmed the glycolytic state of BMDMs by using an extracellular flux assay (Seahorse XFe96). We found that scr-PH-Fib-sEVs induced the extracellular acidification rate in BMDMs, whereas BMDMs treated with siC3-PH-Fib-sEVs exhibited levels similar to those in CO sEV treatment (Figure 7F).

## Discussion

The data of this study demonstrate that sEVs, released by vascular adventitial Fibs derived from PH-Fibs, are critical mediators of complement-driven vascular inflammation that can contribute to the progression of PH. Compared with CO-Fib-sEVs, PH-Fib-sEVs exhibited a markedly distinct proteomic signature that contributed to macrophage activation toward a proinflammatory, metabolically altered phenotype. Moreover, we demonstrate that complement, and specifically the C3 component, was an important determinant of the ability of sEVs to activate macrophages.

PH is a severe, life-threatening cardiopulmonary disorder, wherein inflammation and immunity are the critical early pathogenic determinants (1–6). Although the proinflammatory processes in PH are extensively investigated, the initiating mechanisms remain unclear. Pulmonary vascular Fibs have been recognized to initiate and facilitate recruitment of monocytes/macrophages to perivascular adventitia and their subsequent activation toward a proinflammatory state (2, 9, 13, 34, 39).

A hallmark of vascular inflammation is intercellular communication, and although it can be mediated by both direct cell-to-cell contact and paracrine factors, the local effects of secreted exosomes (EVs) on the target cells is gaining more interest. The role of EVs, which contain proteins and miRNAs and play an important role in cell-to-cell communication, normal homeostasis, and pathology, has been investigated in several tissues, biological fluids, and different cell types (7, 22, 24, 30, 40, 41). However, whether complement-containing EVs play a specific role in vascular inflammatory processes is unknown.

We have recently demonstrated that dysregulated complement activation is an essential pathobiological mechanism regulating early proinflammatory processes in experimental PH, as well a critical determinant of clinical outcome (2, 4, 33). Although the complement system plays a crucial role in protective immune responses, dysregulation of this first line of immune defense can become a potent driver of various inflammatory diseases (2, 32, 33). The mechanisms that initiate and sustain complement signaling in the local tissue microenvironments are complicated and likely involve tissue-specific and cell-specific responses (3, 12, 33, 39). Complement-loaded EVs have been shown to play an important role in a variety of critical cellular processes, including immune, metabolic, and coagulation responses associated with several inflammatory diseases (30, 40–42). Even though numerous studies have explored the role of large vesicles, like EVs, and microvesicles in cardiovascular diseases, little has been reported in the field of PH, especially with regard to a potential role of sEVs in inducing macrophage proinflammatory activation and complement involvement in this process.

In the current study, we employed sEVs isolated from pulmonary vascular adventitial CO-Fibs and PH-Fibs culture to examine their effects on naive BMDMs. Our findings establish that (a) PH-Fibs secreted greater numbers of sEVs compared with CO-Fibs; (b) PH-Fib–derived sEVs mediated BMDM activation toward a proinflammatory and metabolically altered phenotype, wherein the latter was characterized by increased aerobic glycolysis and accumulation of succinate; (c) PH-Fib-sEVs expressed a distinct proteomic signature, compared with CO-Fib-sEVs, with profound enrichment of complement and coagulation components, signifying the induction of proinflammatory and inhibition of antiinflammatory responses; and (d) sEVs derived from PH-Fibs with depleted complement C3 exhibited attenuated stimulation of proinflammatory gene expression and metabolic reprogramming in BMDMs. Collectively, our results suggest that complement-containing sEVs, secreted by pulmonary vascular Fibs from severely hypertensive animals, played a critical role in shaping the local vascular microenvironment in PH.

Differential and density gradient ultracentrifugation are the most commonly used methodologies for isolation of a variety of EVs, including sEVs (22, 25, 42). In our study, to avoid external active complement protein contamination, we used heat-inactivated (thus complement-depleted) and EV-free FBS supplementation in Fib and BMDM in vitro cultures. Thus, this approach allowed an accurate characterization of the sEVs released by Fibs. Our findings demonstrating that PH-Fibs secreted more sEVs into CM than CO-Fibs are similar to studies showing a positive correlation between EV counts and cardiovascular disorders regardless of cell type of origin (43).

It is well established that macrophage phenotypic activation is a process of differentiation into a specific phenotype in response to microenvironment signals, a part of a dynamic reciprocity (12, 15, 18, 44, 45). Our study, establishes that PH-Fib–derived sEVs induced proinflammatory and metabolic reprogramming of BMDMs. Studies by Moon et al. showed that treating alveolar macrophages with hyperoxia-induced sEVs from mouse BALF or type II epithelial cells led to increased macrophage secretion of proinflammatory cytokines, including MIP-2 and IL-6 (46). Macrophages are exceptionally plastic cells and can change their functional profile rapidly. Our data demonstrate that proinflammatory activation of macrophages by PH-Fib-sEVs triggered a metabolic switch toward glycolysis and further away from oxidative phosphorylation, the metabolic gene signature consistent with our recent and other reports (15, 17, 18). Interestingly, treatment of BMDMs with PH-Fib-sEVs also induced the accumulation of succinate, a proinflammatory metabolite that stabilizes hypoxia-inducible factor, thereby promoting transcriptional upregulation of IL-1β (47).

Proteomic analysis of sEVs from Fibs revealed a distinct proteomic signature of PH-Fibs compared with CO-Fibs. GO enrichment analysis of cellular component suggested enrichment of (a) EVs and exosome-related proteins, (b) granule and plasma membrane proteins, (c) cytoskeleton-related proteins, and (d) lipoprotein-related proteins. GO analysis of the molecular function clearly demonstrated the enrichment of immune response and complement activation components. Complement and coagulation cascades were primarily associated with the PH-Fib-sEVs, thus supporting the hypothesis that, in PH, proteins from vascular adventitial Fib-derived sEVs are involved in the activation of macrophage via these pathways. More specifically, network analysis of complement and coagulation cascades suggested induction of proinflammatory responses through upregulation of KNG1 (36, 37) and inhibition of antiinflammatory responses through SERPND1(HCII)–mediated inhibition of F2 (38). Collectively, overrepresented complement and coagulation cascades in PH-Fib-sEVs can act as a “double-edged sword” by both inducing proinflammatory and inhibiting antiinflammatory responses in macrophages.

Complement C3 is a main component of the alternative activation pathway, as well as a central converging component of other (classical and lectin) activation pathways (32). Our data, demonstrating that sEVs from complement C3–depleted PH-Fibs exhibited markedly attenuated proinflammatory responses in BMDMs, suggest that local production of complement C3, loaded into sEVs by pulmonary perivascular Fibs, comprised a sufficient source of complement to induce proinflammatory responses and metabolic reprogramming in macrophages, thus contributing to disease initiation and progression. Even though the liver is the major source of circulating complement proteins, including C3, our findings correspond to a wealth of research demonstrating the role of extrahepatic, local synthesis of complement, and association of the latter with inflammatory and immunological diseases (7, 33, 48–50).

In conclusion, our data necessitate further study on the role of complement in macrophage-mediated tissue inflammation/remodeling and discovery of directed therapeutic approaches to block the progression of PH.

## Methods

### Animals.

The neonatal male Holstein calf model of severe, chronic, hypoxia-induced PH has been described earlier (13, 34). One-day-old calves were purchased from Laluna Dairy Farm, and the experimental group (*n* = 6) was exposed to hypobaric hypoxia (PB = 430 mmHg, simulated elevation 15,000 ft/4570 m) for 2 weeks, whereas age-matched COs (*n* = 6) were kept at ambient altitude (PB = 640 mmHg, 5000 ft/1520 m). Five-week-old WT C57BL/6J male mice were purchased from Jackson Laboratory.

### Cell culture.

Adventitial Fibs from distal pulmonary arteries of 2-week-old male calves were isolated by explant culture as previously described (13, 34) and then cultured with DMEM media containing 4 mM glutamine (Gibco), 25 mM HEPES, 1 mM pyruvate (Gibco), and pen strep 1X (Gibco) supplemented with 10% BCS. All the cells were cultured at 37°C in a 5% CO_2_ humidified environment. All the experiments were performed on cells between passages 6–10. For isolation of sEVs, bovine CO-Fibs (*n* = 6) and PH-Fibs (*n* = 6) were cultured to approximately 70% confluence. Thereafter cells were washed 3 times with PBS (Gibco). EV-depleted fresh DMEM media containing 5 mM glutamine, 25 mM HEPES, and 1 mM pyruvate supplemented with 10% heat-inactivated, EV-depleted FBS was added and culture continued for 48 hours before collection of CM. For isolation of C3-depleted sEVs, we used serum-free medium to avoid C3 contamination. After collection, CM was centrifuged to remove any cell debris, and we used either fresh or aliquots stored at –80°C for further experimental use.

mBMDMs were prepared from 6- to 10-week-old male WT C57B6 mice by incubating extracted bone marrow cells in macrophage growth medium (DMEM containing 5 mM glutamine, 25 mM HEPES, 1 mM pyruvate, and pen strep 1X supplemented with 10% FBS and 50 ng/mL CSF1; BioLegend) for 5–10 days.

Bovine BMDMs were propagated from cells isolated from the ribs of CO neonatal calves. Freshly isolated bovine bone marrow cells were incubated in growth media (10% FBS, DMEM [with pyruvate and 25 mM HEPES], nonessential amino acids, 4 mM Gln, and 50 ng/mL human CSF1) for 9–14 days, until a confluent culture of macrophage cells was achieved.

For biogenesis and secretion inhibition of sEVs, PH-Fibs were treated with 20 μM GW4869 (MilliporeSigma) and cultured for 48 hours followed by washing 3 times with PBS. EV-free fresh DMEM media containing 4 mM glutamine, 25 mM HEPES, and 1 mM pyruvate supplemented with 10% heat-inactivated EV-free FBS was added and further cultured for 48 hours before collection of CM. To remove cells and cell debris, CM was centrifuged at 300*g* for 10 minutes, and supernatants (CM) were used for treatment of mBMDMs.

### Isolation of sEVs.

sEVs were isolated from the CM following a published method (25). CM was subsequently harvested and sEVs were isolated by differential ultracentrifugation methods using serial centrifugation at low speed followed by high-speed ultracentrifugation (Figure 1A). Briefly, CM was centrifuged at 300*g* for 10 minutes at 4°C to pellet cells. Supernatant was centrifuged at 2500*g* for 20 minutes at 4°C, transferred to new tubes, and centrifuged in a 70Ti rotor (Beckman) for 30 minutes at 10,000*g*, then finally for 90 minutes at 100,000*g*. All sEV pellets were washed in 5 mL of PBS and recentrifuged at the same speed before being resuspended in 100–200 μL of sterile EV-free PBS (Gibco). Cells that recovered from the first 300*g* pellet were pooled with cells detached from the plates by incubation at 4°C in trypsin-EDTA (adherent cells; Gibco) and counted by hemocytometer. Concentration and size distribution of particles were measured by NTA. sEV protein was measured by taking OD at 280 nm using a DnNovix DS-11 spectrometer.

### Transmission electron microscopy.

Electron microscopy was performed on freshly isolated sEVs. The sEVs in purified forms were directly loaded on carbon-coated copper grids of 300 mesh and samples were air-dried. The air-dried samples were stained with 1% uranyl acetate for 5 minutes and again the grids were air-dried. These negatively stained grids were imaged by Talos S accelerated at 200 kV at reasonable magnification.

### sEV staining and confocal microscopy.

Freshly isolated sEVs were used for Fib sEV staining. Pellets prepared from ultracentrifugation were suspended in 500 μL of diluent C from the PKH67 kit and 3 μL PKH67 dye (Green Fluorescent Cell linker for General Cell Membrane, MilliporeSigma) was added per the manufacturer’s instructions. Thereafter, the mixture was gently pipetted continuously for 30 seconds and incubated at room temperature for 5 minutes. The reaction was quenched by adding 1 mL 10% BSA in PBS and adjusting the volume to 5 mL in serum-free BMDM media. Stained sEVs were pelleted by ultracentrifugation at 100,000*g* for 90 minutes at 4°C. The pellet was gently resuspended in 50 μL PBS. sEVs were quantitated, and mBMDMs were treated with equal amounts (25 μg/mL) of stained sEVs for 37°C (uptake) and 4°C (CO) for 30 minutes. Cells were mounted with VectaShield/DAPI mounting medium (Vector Laboratories), and images were acquired at 60X using Zeiss LSM780 spectral. Images were processed with ImageJ Fiji software.

### Activation of mBMDMs.

For mBMDM activation experiments, 0.5 million mBMDMs were plated per well of a 24-well plate in the macrophage growth medium. Media of mBMDMs were replaced with fresh EV-free, complement-inactivated DMEM media (normal FBS replaced with 10% heat-inactivated, EV-depleted FBS) and treated with optimal doses of sEVs (25 μg sEVs protein/mL ~10^9^ sEVs/mL) derived from CO-Fibs (*n* = 3 or 6) or PH-Fibs (*n* = 3 or 6) and incubated for 16 hours. We used equal amounts CO-Fib-sEVs or PH-Fib-sEVs for treatment of BMDMs. We used the same amount of sEVs as in the CM for the initial evaluation of mBMDM activation (Figure 2A).

### Quantitative real-time PCR.

Total RNA of cultured cells was extracted using the NucleoSpin RNA purification kits (Machery-Nagel) per the manufacturer’s instructions. RNA quality and quantity were analyzed using a Denovix DS-11 spectrophotometer. First-strand cDNA synthesis was performed using an iScript cDNA Synthesis Kit (Bio-Rad). Quantitative real-time PCR was performed using either TaqMan probes (Applied Biosystems) or SYBR Green supermax (Bio-Rad) with ABI-7500 Real Time PCR System (Applied Biosystems). All probes are listed in Supplemental Table 3. Gene expression was calculated after normalization to *Hprt* using the relative quantitative ΔΔCt method, and the fold change was calculated relative to the COs in each group.

### Mouse cytokine array analysis.

Mouse cytokine array was performed per the manufacturer’s guidelines (Proteome Profiler Array, R&D Systems). In brief, CM from mBMDMs treated with CO-Fib-sEVs and PH-Fib-sEVs were collected. CM (600 μL) from each sample with the appropriate amount of binding buffer were mixed and incubated overnight (16 hours) with a membrane containing 40 antibodies. Membranes were exposed to x-ray film and fixed and developed in respective solutions. Developed film was scanned and the estimated intensity of each dot was quantified by NIH ImageJ software.

### IL-1B ELISA.

Cell culture supernatant was collected from mBMDMs after the 16-hour incubation period, and IL-1B was quantified using a mouse IL-1B ELISA (R&D Systems) per the manufacturer’s protocols.

### Cell transfection.

Transfection was performed with 50 nmol/L of miR-C3 mimic, scramble, or siRNA targeting C3 (Supplemental Table 3) using DharmaFECT transfection reagents (Dharmacon Inc.) per the manufacturer’s instructions. One set of the cells was harvested for mRNA expression after 48 hours of transfection and another set for isolation of C3-depleted sEVs.

### Metabolomics.

BMDMs (0.5 × 10^6^ cells) were incubated with either PBS or sEVs derived from CO-Fibs or PH-Fibs (*n* = 4 per group), in the presence of stable isotope-labeled U-^13^C_6_-glucose in culture for 1 hour and 4 hours. Cells were extracted in methanol/acetonitrile/water (5:3:2 v/v/v) prior to UHPLC-MS analyses (Vanquish-QExactive, Thermo Fisher Scientific) as previously described (17, 51). For global metabolomics studies, BMDMs (0.5 × 10^6^ cells) were incubated with scr-PH-Fib-sEVs (*n* = 4) and siC3 PH-Fib-sEVs (*n* = 4) for 16 hours; extracts were obtained and then processed with the abovementioned method.

### Proteomics and mass spectrometry analysis.

Proteomics analyses were performed with fresh frozen CO-Fib-sEVs (*n* = 3) and PH-Fib-sEVs (*n* = 4). The sEV protein samples were digested according to the filter-aided sample preparation protocol using a 10 kDa–molecular weight cutoff filter. Protein digestion was carried out with sequencing grade modified Trypsin (Promega) at 1:50 protease/protein (w/w) at 37°C overnight. Peptides were recovered from the filter using 50 mM AB. Samples were dried in Speed-Vac, desalted, and concentrated on Thermo Scientific Pierce C18 Tips. Digested samples were analyzed on an Orbitrap Fusion mass spectrometer (Thermo Fisher Scientific) coupled to an Easy-nLC 1200 system (Thermo Fisher Scientific) through a nanoelectrospray ion source. MS/MS spectra were extracted from raw data files and converted into mgf files using a Proteome Discoverer software (version 2.1.0.62). Subsequent database searching against a bovine uniprot fasta file was performed using the Mascot algorithm (version 2.6, Matrix Sciences). Label-free quantification was performed using Scaffold (version 4.8, Proteome Software). Peptide identifications were accepted if they could be established at greater than 95.0% probability as specified by the Peptide Prophet algorithm.

### MS data processing.

Data were filtered when less than 50% complete across samples and normalized by total signal intensity. Limma package of R was used for differential expressed protein analysis, and ggplot2 (52) was used to construct PCA to see variation within and between the groups. For statistics verification, unpaired *t* test with Welch’s correction was used to analyze differences between PH-Fib-sEVs and CO-Fib-sEVs, and a *q* value of less than 0.2 was considered statistically significant. Heatmap and volcano plots of expressed protein were generated using the Bioconductor R package ComplexHeatmap (52) and ggplot2, respectively. GO was performed by EnrichmentMap (53) and BiNGO (54) tools of Cytoscape v3.8.2 (55), and pathway enrichment analysis was performed by web-based bioinformatics tools at Enrichr (https://amp.pharm.mssm.edu/Enrichr/), searching in the KEGG, Wikipathway, and Reactome databases (56). A network of pathways was generated and visualized by wikipathway and KEGG network of Cytoscape tools.

### Measurement of extracellular acidification rate and oxygen consumption rate by extracellular flux analysis.

The Seahorse Extracellular Flux analyzer (XFe96) was used to evaluate the glycolysis by measurement of extracellular acidification rate and mitochondrial respiration by measurement of oxygen consumption rate of BMDMs.

### Statistics.

Bioconductor R package ggplot2 and ggpubr were used for plotting the graphs and statistical analysis; values were expressed as mean ± SEM. Unpaired 2-tailed *t* test with Welch’s correction was used to compere 2 group of samples. For more than 2 groups with 1 variable, 1-way ANOVA was performed. For more than 2 groups with 2 independent variables, 2-way ANOVA followed by Tukey’s HSD post test was performed. Shapiro-Wilk test for normality and Levene’s test for homogeneity of variance across groups by using car package (R) were used to assess for normality before applying parametric statistical tests. Nonparametric test was performed if data did not pass the parametric assumption. Values were expressed as mean ± SEM. Differences with *P* values less than 0.05 were considered statistically significant.

### Study approval.

Standard husbandry and veterinary care of neonatal calves were provided following institutional guidelines at the Hypobaric/Hyperbaric Facility, Department of Physiology, Colorado State University (Fort Collins, Colorado, USA). Standard veterinary care was provided, and all animal procedures were performed in accordance with the instructions for animal experimentation established and approved by the IACUC of the University of Colorado Anschutz Medical Campus.

**Author contributions**

SK conceived the study, designed and performed the experiments, statistically analyzed and interpreted the data, made the figures, and wrote the manuscript. MGF, HZ, ML, SR, RDB, SCY, MED, and MKR performed the experiments and analyzed the data. MGF and HZ edited the manuscript. KRS, AD, and KCH provided intellectual oversight of the project, interpreted the data, and edited the manuscript.

## Supplementary Material

Supplemental data

Supplemental table 1

Supplemental table 2

Supplemental table 3

## Version 1 09/09/2021

In-Press Preview

## Version 2 11/08/2021

Electronic publication
