# Supplementary material for: Complement-containing small extracellular vesicles from adventitial fibroblasts induce proinflammatory and metabolic reprogramming in macrophages
Source: JCI Insight. 2021 Nov 8;6(21):e148382. doi: 10.1172/jci.insight.148382 (PMC8663554; doi:10.1172/jci.insight.148382)
Supplement: Supplemental data [file jciinsight-6-148382-s147.pdf]

Supplement figure 1

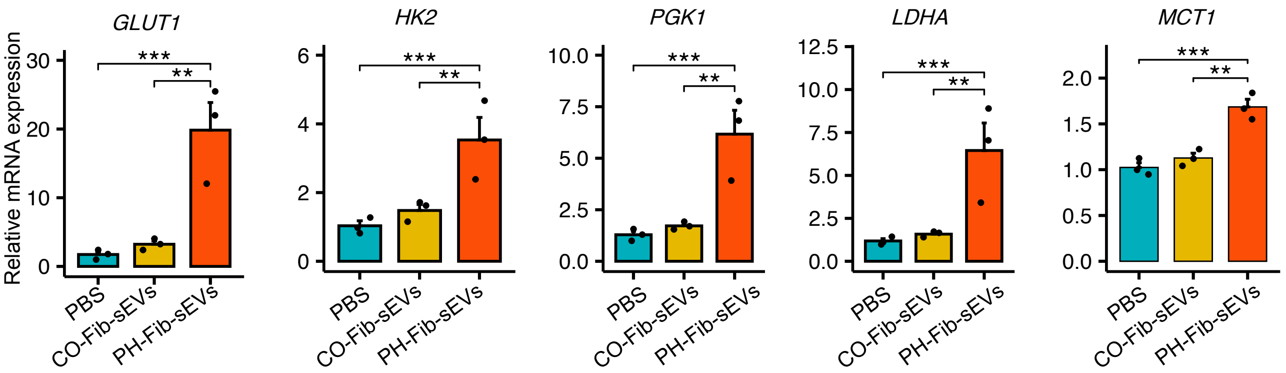

Supplement figure 1. sEVs derived from PH-Fibs induce changes in the expression of metabolic genes inbovine BMDMs. sEVs from PH-Fibs induce expression of GLUT1, HK2, PGK1, LDHA, and MCT1 in bovineBMDM. Data presented (n=3 each group) as mean ± SE. One-way ANOVA followed by Tukey's multiplecomparisontest were performed. *denotes $p < 0.05$; **denotes $p < 0.01$; ***denotes $p < 0.001$.

Supplement figure 2

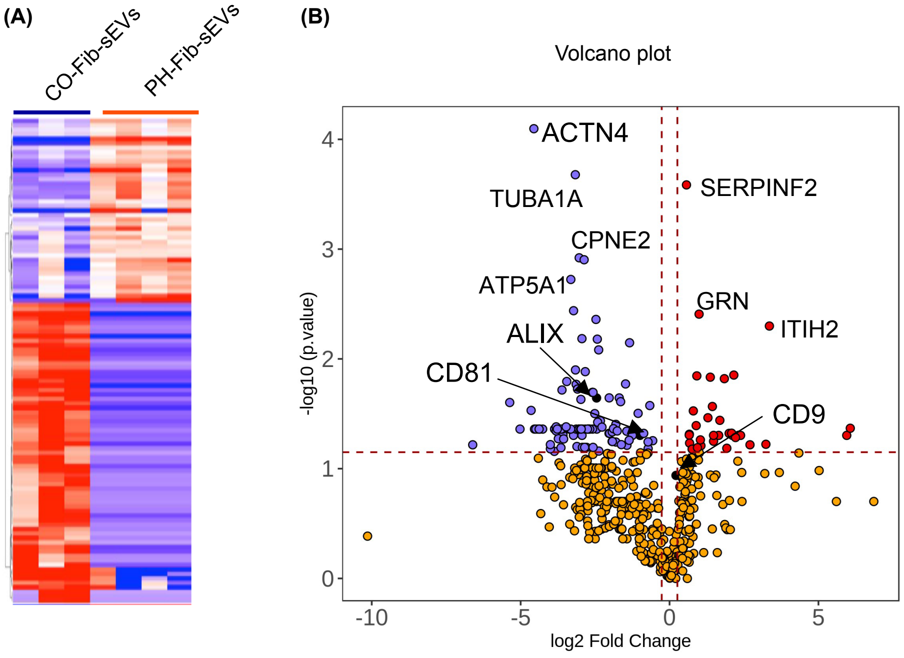

Supplement figure 2. Proteomic analysis of sEVs from CO-Fibs and PH-Fibs shows broad and distinct proteomic signatures. (**A**) A heatmap showing the differentially expressed proteins between the two groups and (**B**) volcano plot showing differentially expressed proteins. The X-axis represents the log2 fold change and the Y-axis represents the –log10(p-value). Horizontal dashed line represents p-value = 0.05 cut off and 2 vertical dashed line represent fold change cut off (±1.2 FC). Data presented, CO-Fibs (n=3) and PH-Fibs (n=4). Unpaired t-test with Welch's correction were used for comparison between two groups. p < 0.05 considered as differentially expressed protein.

Supplement figure 3

(A) Enrichment of Cellular Component gene ontology in PH-Fib-sEVs

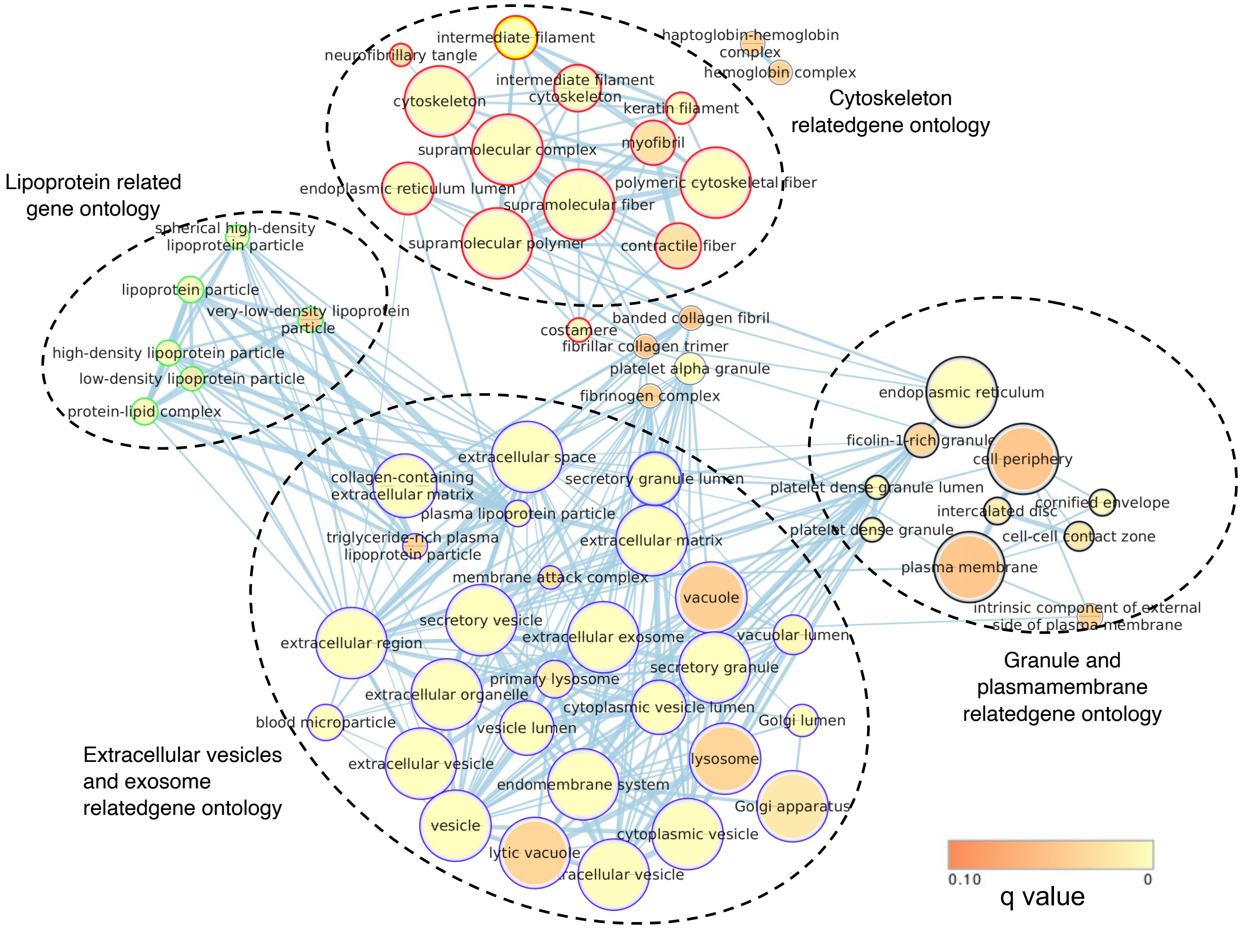

(B) Enrichment of Molecular Function gene ontology in PH-Fib-sEVs

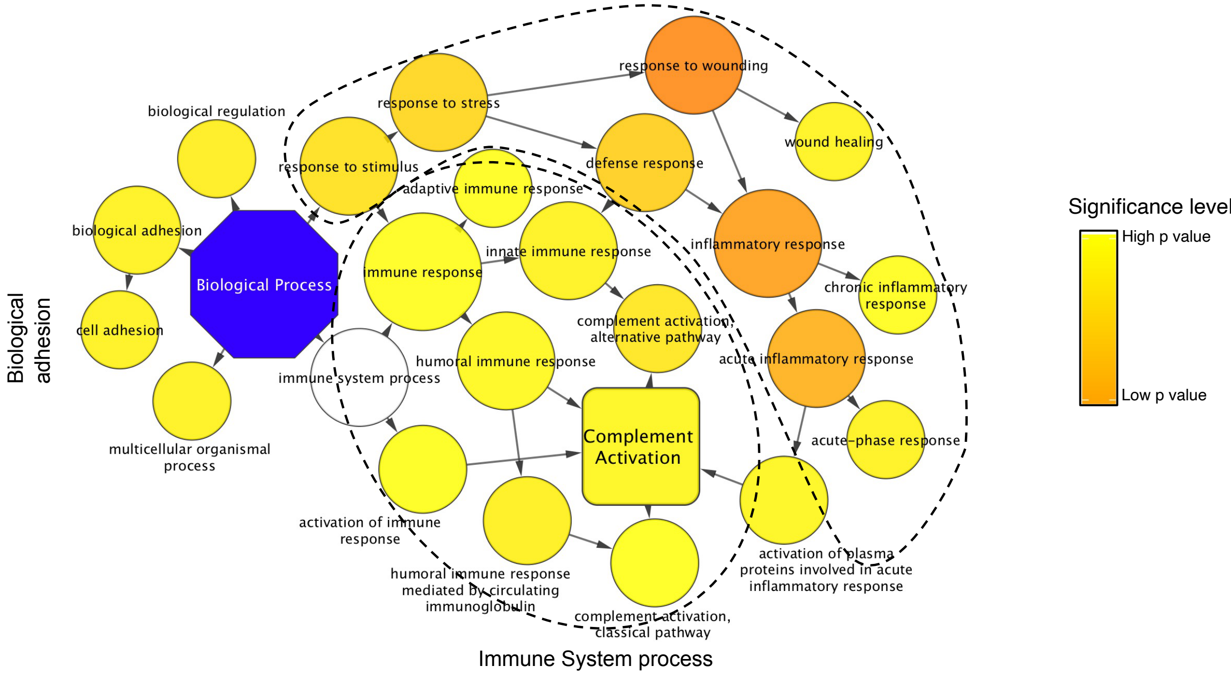

Supplement figure 3. Gene Ontology (GO) enrichment analysis of differentially expressed sEVs proteins. (A) The cellular component (CC) of differentially expressed sEVs proteins revealed enrichment of extracellular vesiclesand exosome, granule and plasma membrane, cytoskeleton, and lipoprotein related gene ontology in PH-Fibs. **(B)**The biological process (BP) of differentially expressed sEVs protein revealed enrichment of immune response,response to wounding, inflammation response, biological regulation, biological adhesion, including activation ofcomplement. Size of sphere corresponds to number of proteins. The color gradient of the cluster distribution networkshows the p-value and q-value of each cluster associated with the GO term.

Supplement figure 4 Fold-change and p value: PH scr vs PH siC3

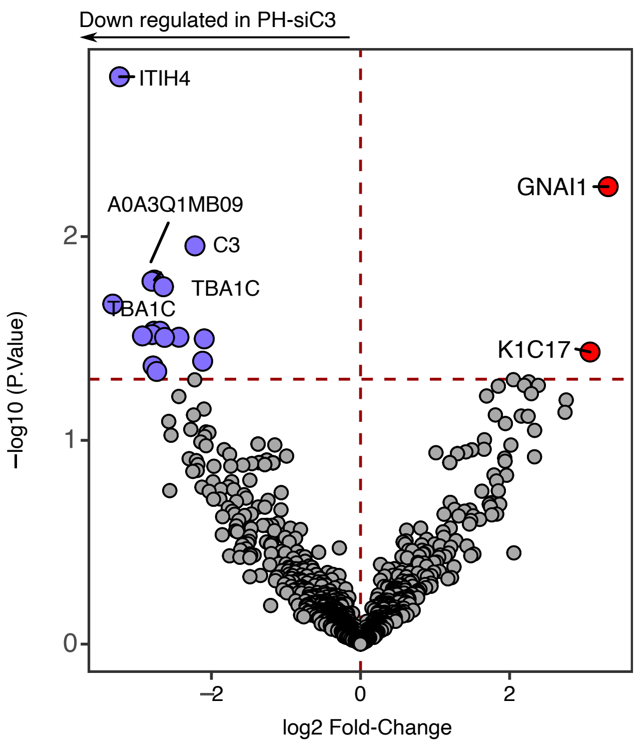

Supplement figure 4. C3 protein depletion in PH-Fibs sEVs. Volcano plot of differentially change protein of C3 depleted sEVs represents significant downregulation of C3 in PH-siC3 sEVs. The protein shown in the upper left compartments, represent those that are significantly downregulated, while the sEVs protein, shown in the upper right compartments, represent those that are significantly upregulated.
